# Deep Learning for the Automatic Segmentation of Extracranial Venous Malformations of the Head and Neck from MR Images Using 3D U-Net

**DOI:** 10.3390/jcm11195593

**Published:** 2022-09-23

**Authors:** Jeong Yeop Ryu, Hyun Ki Hong, Hyun Geun Cho, Joon Seok Lee, Byeong Cheol Yoo, Min Hyeok Choi, Ho Yun Chung

**Affiliations:** 1 Department of Plastic and Reconstructive Surgery, School of Medicine, Kyungpook National University, Daegu 41944, Korea; 2 DEEPNOID Co., Seoul 08376, Korea; 3 Cell & Matrix Research Institute, School of Medicine, Kyungpook National University, Daegu 41944, Korea

**Keywords:** vascular malformations, deep learning, surgery, plastic

## Abstract

Background: It is difficult to characterize extracranial venous malformations (VMs) of the head and neck region from magnetic resonance imaging (MRI) manually and one at a time. We attempted to perform the automatic segmentation of lesions from MRI of extracranial VMs using a convolutional neural network as a deep learning tool. Methods: T2-weighted MRI from 53 patients with extracranial VMs in the head and neck region was used for annotations. Preprocessing management was performed before training. Three-dimensional U-Net was used as a segmentation model. Dice similarity coefficients were evaluated along with other indicators. Results: Dice similarity coefficients in 3D U-Net were found to be 99.75% in the training set and 60.62% in the test set. The models showed overfitting, which can be resolved with a larger number of objects, i.e., MRI VM images. Conclusions: Our pilot study showed sufficient potential for the automatic segmentation of extracranial VMs through deep learning using MR images from VM patients. The overfitting phenomenon observed will be resolved with a larger number of MRI VM images.

## 1. Introduction

Venous malformations (VMs) are caused by abnormalities in vascular morphogenesis. Vascular anomalies are classified into vascular tumors and vascular malformations based on Mulliken’s findings and according to the International Society for the Study of Vascular Anomalies [1,2]. Vascular malformations are differentiated into simple, combined malformations, vascular malformations of major named vessels, and those associated with other anomalies. Among simple vascular malformations, VMs are the most common ones [2,3]. VMs are known to occur most often in the head and neck region (47%) [4].

**Figure 1:**
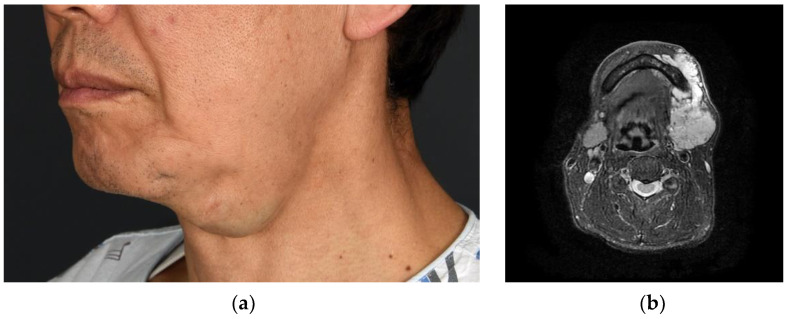
VM in the left submandibular area. (**a**) Bulging blueish vascular mass was observed; (**b**) MRI findings of VM. These T2-weighted with fat suppression MRI findings showed an infiltrative vascular channel with a size of 8.2 × 4.1 cm from the subcutaneous fat layer to the submandibular gland and part of the posterior belly of the digastric muscle in the left submandibular area. It showed inhomogeneous patchy high signal intensity inside. VM, venous malformation; MRI, magnetic resonance imaging.

In the diagnosis of VMs from images, B-mode sonography, in combination with color-coded duplex sonography (CCDS) and magnetic resonance imaging (MRI), is often used [3,5]. CCDS is the first imaging modality in the diagnosis of vascular malformations. It gains morphologic information in addition to information about blood flow. CCDS is also suitable for the depiction of flow characteristics. MR images depict the extents of lesions and their relationships with surrounding structures. MRI findings of VMs are seen as hyperintense lesions in T2-weighted fat-saturated sequence images as well as hypointense or isointense lesions in T1-weighted images without a contrast medium relative to muscle (Figure 1) [6]. However, although single-mass VMs appear as single-mass-like lesions in MRI, VMs in many patients are seen as multifocal lesions over several areas in the head and neck region. Significant time and effort are needed for clinicians to determine the boundaries of lesions in multifocal areas one at a time and to calculate their volumes. Recently, the development and use of automatic segmentation using convolutional neural networks (CNNs) as a deep learning tool has been reported in many fields, but the segmentation of blood vessels continues to be challenging [7,8,9,10,11].

We attempted to utilize an artificial intelligence (AI) strategy to automatically determine the boundaries of lesions and perform segmentation to distinguish lesions from other tissues through deep learning using MRI of VMs.

## 2. Materials and Methods

### 2.1. The Dataset and Preprocessing

According to thorough examinations in the multidisciplinary Vascular Anomalies Center, 53 patients were diagnosed with VMs of the head and neck region and routinely subjected to MRI. In these MR images, all of the images were taken from the neck to the vertex of the head. Two plastic surgeons with 20 and 10 years of experience in the field of vascular anomalies, respectively, performed data annotation using T2-weighted images with fat suppression of 3D MRI datasets in accordance with the readings of radiological specialists for vascular anomalies. All MR images were taken using 3.0T SIGNA™ MRI Scanners (GE HealthCare, Waukesha, WI, USA). In the T2-weighted axial image with fat suppression, the boundary between hyperintense lesions and non-hyperintense normal tissues was drawn using the labeling software DEEP:LABEL^®^ (DEEPNOID Inc, Seoul, Korea). For accurate data labeling, they drew the boundary between the lesion and the healthy surrounding tissue directly on the screen using a touchscreen computer and a Bluetooth pen. In the preprocessing of the images, all of the images, of various dimensions, were resized to a volume of 16, a height of 128, and a width of 128 pixels. After resizing, the images were inverted and contrast-limited adaptive histogram equalization (CLAHE) was applied to effectively improve the image contrast in cases of light and dark areas in the images. In order to make the dynamic range of image processing consistent and increase the efficiency of calculations, a Z-score was standardized. The formula used for Z-score standardization was $Z=\frac{x-\mu }{\sigma }$ (x, pixel values of the original image; *µ*, mean pixel value of the original image; σ, pixel standard deviation of the original image). As the last step of preprocessing, minimum–maximum normalization was performed. This unified the pixel values of all images so that the CNN did not learn unnecessary features. The formula used for normalization was $X_{normalization}=\frac{X-X_{min}}{X_{max}-X_{min}}$. Thus, the image pixel value was set to 0–1. All of the preprocessing procedures were performed using a research platform for machine learning called DEEP:PHI^®^ (DEEPNOID Inc., Seoul, Korea), which is available to everyone. More details on the platform can be found on its official website (https://www.deepphi.ai, accessed on 23 September 2022).

### 2.2. CNN Architecture and Performance Analysis

Three-dimensional U-Net was used as the CNN architecture [12]. U-Net is a CNN frequently used for image segmentation, and its performance has been verified in various medical image deep learning studies [13,14]. By including the skip connection technique in the general encoder–decoder structure, the information before the feature map is compressed in the convolution layer, saved, and sent to the decoder, which results in better performance than that of fully convolutional networks [15]. In the original U-Net, segmentation was performed with 2D-based images, but the dataset used in our study comprised 3D MR images. Therefore, we used 3D U-Net that replaced the 2D convolution layer with a 3D convolution layer in the original U-Net structure [11]. Among the 53 patients with VMs in the head and neck region, MRI of 48 patients was used as the training set and that of 5 patients was used as the test set. MRI segmentation was conducted using different key performance measures, including sensitivity, specificity, accuracy, positive predictive value (PPV), negative predictive value (NPV), and Dice similarity coefficient. The formula for each measurement was as follows:

$$Sensitivity=\frac{TP}{TP+FN}$$

$$Specificity=\frac{TN}{FP+TN}$$

$$Accuracy=\frac{TP+TN}{TP+FN+FP+TN}$$

$$PPV=\frac{TP}{TP+FP}$$

$$NPV=\frac{TN}{TN+FN}$$

$$Dice\;similarity\;coefficient=\frac{2\times TP}{2\times TP+FP+FN}$$

*TP*, true positive; *TN*, true negative; *FP*, false positive; *FN*, false negative; *PPV*, positive predictive value; and *NPV*, negative predictive value.

## 3. Results

We retrospectively reviewed 53 patients who were diagnosed with venous malformations in the head and neck region. The mean age of the 53 patients (28 of which were female) was 26 years (4–70 years). Thirty-six of the patients had localized solitary lesions, while seventeen of the patients had multifocal lesions. Ten lesions were distributed in the upper face (eyebrow to vertex), thirty-three were in the midface (lip to eyebrow), and twenty-eight were in the lower face and neck. The reason the total number of lesions exceeded 53 was because multifocal lesions were counted for every single VM manifestation. Detailed characteristics of the study population are summarized in Table 1.

The Dice similarity coefficient, the most important index in image segmentation, showed a training set performance of 99.75% and a test set performance of 60.62%. Other performance indicators are reported in Table 2, showing very high performance in the training set and relatively low performance in the test set.

### 3.1. Cases: Data of the Training Set

**Figure 2:**
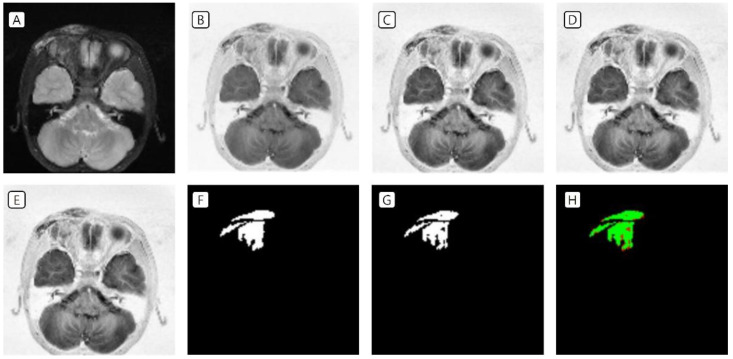
Training set of VMs in the right periorbital area. (**A**) Resized to a volume of 16, a height of 128, and a width of 128 pixels; (**B**) inverted image; (**C**) CLAHE application; (**D**) Z-score standardization; (**E**) minimum–maximum normalization; (**F**) labeling data drawn by the clinicians; (**G**) prediction data drawn by the artificial intelligence tool; and (**H**) green indicates the matched area, while red indicates the mismatched area. VMs, venous malformations; CLAHE, contrast-limited adaptive histogram equalization.

**Figure 3:**
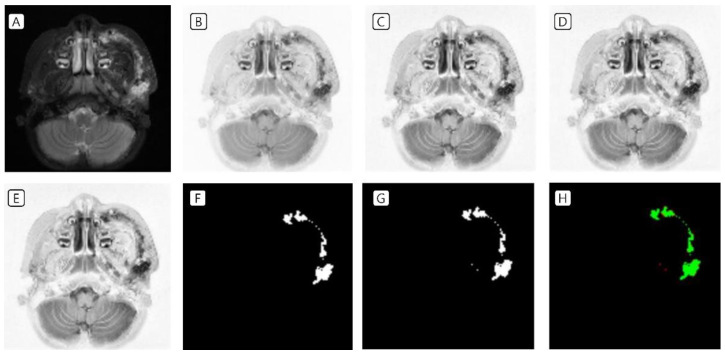
Another training set of VMs in the left cheek. (**A**) Resized to a volume of 16, a height of 128, and a width of 128 pixels; (**B**) inverted image; (**C**) CLAHE application; (**D**) Z-score standardization; (**E**) minimum–maximum normalization; (**F**) labeling data drawn by the clinicians; (**G**) prediction data drawn by the artificial intelligence tool; and (**H**) green indicates the matched area, while red indicates the mismatched area. VMs, venous malformations; CLAHE, contrast-limited adaptive histogram equalization.

T2-weighted MRI of patients with VMs in the head and neck region was used as data in the training set. In the image preprocessing, the image was (A) resized to a volume of 16, a height of 128, and a width of 128 pixels; (B) the image was inverted; (C) CLAHE was applied; and (D) Z-score standardization as well as (E) minimum–maximum normalization were performed. The labeling data drawn by the plastic surgeon (F) and the prediction data drawn by the AI tool (G) were almost identical (Figure 2 and Figure 3).

### 3.2. Cases: Data of the Test Set

**Figure 4:**
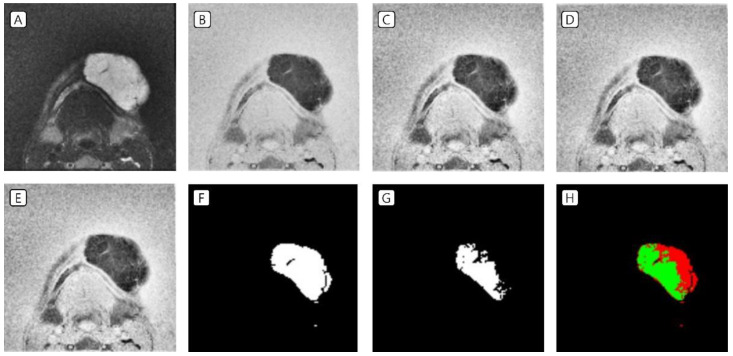
Test set of VMs in the left mandibular area. (**A**) Resized to a volume of 16, a height of 128, and a width of 128 pixels; (**B**) inverted image; (**C**) CLAHE application; (**D**) Z-score standardization; (**E**) minimum–maximum normalization; (**F**) labeling data drawn by the clinicians; (**G**) prediction data drawn by the artificial intelligence tool; and (**H**) green indicates the matched area, while red indicates the mismatched area. VMs, venous malformations; CLAHE, contrast-limited adaptive histogram equalization.

**Figure 5:**
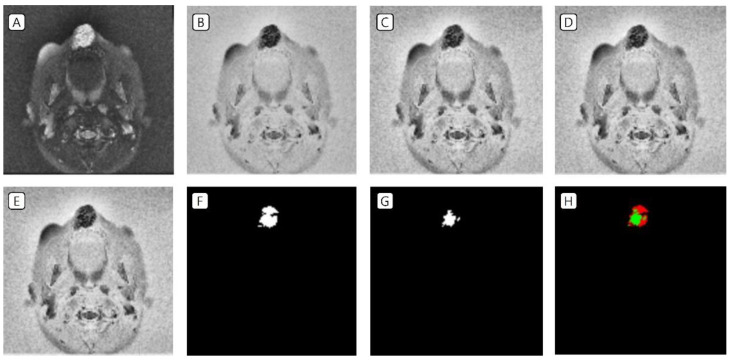
Another test set of VMs in the lower lip. (**A**) Resized to a volume of 16, a height of 128, and a width of 128 pixels; (**B**) inverted image; (**C**) CLAHE application; (**D**) Z-score standardization; (**E**) minimum–maximum normalization; (**F**) labeling data drawn by the clinicians; (**G**) prediction data drawn by the artificial intelligence tool; and (**H**) green indicates the matched area, while red indicates the mismatched area. VMs, venous malformations; CLAHE, contrast-limited adaptive histogram equalization.

T2-weighted MRI of patients with VMs in the head and neck region was used as data in the test set. In the image preprocessing, the image was (A) resized to a volume of 16, a height of 128, and a width of 128 pixels; (B) the image was inverted; (C) CLAHE was applied; and (D) Z-score standardization as well as (E) minimum–maximum normalization were performed. The labeling data drawn by plastic surgeons (F) and the prediction data drawn by the AI tool (G) were identical in some areas (Figure 4 and Figure 5).

## 4. Discussion

VMs in the head and neck region are known for being difficult to treat [3,16]. Their treatment includes sclerotherapy and excisional surgery, with sclerotherapy being more preferred due to the concentration of important structures, such as nerves and the orbits, in this anatomical region. Furthermore, patients favor minimal invasive treatment without subsequent cosmetic impairment [3,17]. For the accurate diagnosis of VMs in the head and neck region, MR images are important for (1) confirming the diagnosis, (2) specifying the extent of the VMs, and (3) making a treatment plan [4]. However, it is not easy to specify and quantitatively analyze lesions in MRI. Therefore, we performed a study to automatically measure the volume of localized VMs as well as isolated lesions of multifocal disease on MR images using CNN architecture as an AI tool. The use of a CNN for medical image recognition has been widely used. Among them, U-Net is used for automatic segmentation and has been applied to differentiate between breast and fibroglandular tissue from breast MRI, wound regions from healthy skin on photographs, and spontaneous intracerebral hemorrhages from unaffected brains in computed tomography images [18,19,20,21]. It has also been applied to cell detection and segmentation, providing evidence that U-Net yields results comparable in quality to manual annotation [22].

In the present study, preprocessing, including resizing, inversion, CLAHE, Z-score standardization, and minimum–maximum normalization, was implemented. Generally, the inversion of MR images is necessary as there is a tendency for CNNs to focus on the brightest area in the image. In this study, the algorithm initially often mistakenly segmented the brain parenchyma for a lesion during machine learning due to the fact that the VM manifestations as well as brain parenchyma were hyperintense in the original data. After inversion, the results improved clearly. In spite of efforts to improve this performance, the Dice similarity coefficient of the training set showed clearly better performance than the test set did (99.75% vs. 60.62%). Thus, the error in the test set was higher than that of the training set. This is due to “overfitting” [23]. Overfitting refers to a phenomenon in which the model is overfitted to the training data and does not operate accurately outside the training data and does not generalize. There are two main reasons for the occurrence of overfitting: model complexity and insufficient training data. We used 3D U-Net as a segmentation model in our study, which has been applied successfully in several deep learning studies. U-Net is widely used because it has good prediction performance for the region of interest and the details of the background. However, as a limitation, the accuracy decreases when the size of the lesion is small. Recently, neural networks with advanced U-Net architecture are also used to overcome these limitations [24]. The other possible cause is the use of insufficient MRI data for model training, which is more likely to be the case here. The most intuitive way to solve this problem is to train the model with sufficiently large datasets. This also suggests that the problem of overfitting in our model will be solved as the amount of data used increases. The performance of the machine improves as more training datasets are added to the machine learning process. However, it is difficult to quantitatively know how many additional datasets are necessary. Hu et al. reported a concept of model complexity of deep learning [25]. In addition, as a recent trend, active learning is sometimes used in the process of upgrading performance. This is a way to improve performance by adding datasets similar to specific datasets that neural networks struggle with. The active learning methodology seems to achieve a larger improvement in performance with the same or fewer numbers of datasets compared to the performance generally obtained by adding datasets [26].

VMs represent a challenging condition, especially when located in the head and neck region. The main approaches for treating VMs are sclerotherapy and surgical resection [3,5]. The evaluation of treatments is also performed using MRI or CCDS, as well as clinical features. To evaluate lesion size as one of indicators of therapy effectiveness, it is necessary to calculate the volumes of lesions from MRI. However, VMs may appear in several places, and there may be cases with no clear lesion boundaries, making it difficult to calculate lesion volumes one at a time. Therefore, it would be very convenient to develop a system that automatically quantifies lesions in MRI; to accomplish this, it is necessary to first segment lesions automatically through deep learning. Next, the segmented area and the integral calculus are automatically calculated to arrive at the lesion volume. If the machine learning is carried out by including data with various factors affecting the prognosis and recurrence rate, such as surgery vs. sclerotherapy, type of sclerosing agent, dose of the agents, location of the lesion, preoperative and postoperative volumes of lesions, etc., we think that it will be possible to predict a patient’s prognosis when the regression models are developed. In particular, it is difficult to determine whether sclerotherapy or surgical resection is better as a treatment for VMs in the head and neck region. Because treatment decisions differ in each case, it would be of great help in clinical decision making if AI could predict the prognosis of sclerotherapy or surgical resection in each case.

## 5. Conclusions

The present study implemented a model that automatically segmented MRI of VMs using deep learning. Overfitting resulted in high performance with the training set and low performance with the test set, and this is expected to be resolved with larger VM MRI datasets. Performance in the automatic segmentation of VMs from MRI showed sufficient potential. The automatic segmentation of VMs would also allow the calculation of VM volumes, and, with further research, potentially catalyze progress toward the ultimate goal of the prediction of prognoses following VM treatments.

## Figures and Tables

**Table 1:** Patient characteristics of the target dataset.

Variables	Value
Number of patients	53
Mean age	26 (4–70)
Female sex	28 (52.83%)
Multifocal lesions	17 (32.08%)
Distribution of lesions	
Upper face	10 (14.08%)
Midface	33 (46.48%)
Lower face to neck	28 (39.44%)

**Table 2:** Results of automatic segmentation using 3D U-Net.

Performances (%)	Training Set	Test Set
Sensitivity	99.75	45.10
Specificity	100	99.96
Accuracy	100	99.43
PPV ^1^	99.74	92.45
NPV ^2^	100	99.47
Dice ^3^	99.75	60.62

^1^ PPV, positive predictive value; ^2^ NPV, negative predictive value; and ^3^ Dice, Dice similarity coefficient.

## Data Availability

The data presented in this study are available on request from the corresponding author. The data are not publicly available due to personal information protection principles.
